# The effect of artificial intelligence-empowered mobile health on psychological distress in women following abortion: protocol for a mixed-methods study

**DOI:** 10.3389/fpsyt.2025.1665500

**Published:** 2025-11-18

**Authors:** Wei Zhang, Yuqi Yang, Meimei Liu, Lirong Wang, Qing Lei, Qiumei Zhang, Jing Wang, Hui Li, Gumula Wuri

**Affiliations:** 1 Department of Gynecology, Inner Mongolia Maternity and Child Health Care Hospital, Hohhot, Inner Mongolia, China; 2 School of Nursing, Henan University of Science and Technology, Luoyang, Henan, China; 3 School of Public Health, Peking University, Beijing, China; 4 Nursing Department, Shandong Provincial Hospital Affiliated to Shandong First Medical University, Jinan, Shandong, China

**Keywords:** artificial intelligence, mobile health, Swanson’s theory of caring, large language model, abortion, psychological distress, mixed-methods

## Abstract

**Background:**

The number of abortions worldwide continues to rise, and abortion can have adverse physical and psychological effects. At present, there is little attention paid to the psychological distress experienced by women after abortion, such as anxiety and depression. Current interventions rely heavily on specific personnel, time, and location, which can be costly. Artificial Intelligence-Empowered Mobile Health can compensate for the shortcomings of current interventions, and these interventions are guided by Swanson’s Theory of Caring.

**Methods:**

This study is a mixed-methods research protocol to be implemented at the Inner Mongolia Maternity and Child Health Care Hospital. A total of 100 women after abortion will be included and randomly assigned to either the intervention group or the standard care group. The intervention group will receive two weeks of Artificial Intelligence-Empowered Mobile Health intervention guided by Swanson’s Theory of Caring, including medical knowledge about abortion, post-operative diet, and exercise guidance. The standard care group will receive standard prenatal care. The primary outcome is the change in depression levels, while secondary outcomes include anxiety, perceived stress, perceived social support, and other factors. This study uses IBM SPSS Statistics 27.0 and NVivo 12.0 for data analysis, employing descriptive statistics, normality tests, Mann–Whitney U, Wilcoxon, and chi-square tests.

**Discussion:**

This study pioneers an Artificial Intelligence-Empowered Mobile Health guided by Swanson’s Theory of Caring, providing continuous post-abortion support to reduce psychological distress. It applies Large Language Models to Artificial Intelligence-Empowered Mobile Health for women experienced abortion, delivering timely, specialized care. This approach overcomes traditional barriers: offering real-time interaction, breaking spatiotemporal limits, lowering costs, and integrating expert knowledge to mitigate regional resource disparities, and also promoting health equity.

## 1 Background

Miscarriage is generally defined as the loss of a pregnancy before viability. Abortion is a simple health care intervention that can be effectively managed by health workers through medication or surgery. Medical abortion involves using drugs to reduce the biological activity of progesterone and then inducing strong uterine contractions to expel the pregnancy tissue from the body. The drugs commonly used in clinical practice are mifepristone and misoprostol (1). Surgical abortion involves removing the contents of the uterus through the vagina. Different techniques are used depending on the length of pregnancy. Common techniques include manual or electric vacuum aspiration and hysteroscopy (2).

Approximately 73 million abortions are performed worldwide each year. Six out of ten (61%) of all accidental pregnancies and three out of ten (29%) of all pregnancies are terminated by abortion (3). About 10.8% of women experience at least one miscarriage in their lifetime, 1.9% experience two, and 0.7% experience three or more (4). It is estimated that there are about 1.034 million abortions in the United States each year (5), while the total number of abortions in China is estimated to be about 13 million (including cases not fully counted by private hospitals and clinics). Furthermore, since 2017, the number of abortions in China has been increasing annually (6).

Insecure abortion procedures or improper postoperative care can easily lead to hemorrhage and infection. In developing countries, unsafe abortions are one of the main direct causes of maternal mortality (7). Abortion can also affect future pregnancies. Women with a history of abortion have an increased risk of premature delivery and are more likely to experience placental abnormalities (such as placenta praevia, placental abruption, etc.) (4).

Abortion can also have adverse psychological effects on women. Women may experience post-traumatic stress disorder, stress, anxiety, and depression after an abortion (8). Over 60% of interviewees reported occasional or frequent depression and anxiety after abortion (9). Moreover, these psychological distress symptoms persist for longer periods, lasting 14 to 31 months after the abortion (10). After an abortion, women may feel stigmatized due to societal controversy, leading to feelings of guilt, self-blame, or shame, which may exacerbate symptoms of depression, trauma, and anxiety (11).

Abortion costs affect individuals, the healthcare system, and society. It is estimated that the short-term economic loss caused by abortion in the UK each year amounts to £471 million (4). Complications arising from unsafe abortions require a higher level of care (12), which not only increases treatment costs, leading to financial losses for individuals and consumption of medical resources (13), but also causes labor losses (14). In addition, abortion bans in some countries and regions will increase public expenditure, such as medical subsidies (15).

Current attention to psychological distress after abortion is insufficient. Routine intervention relies heavily on medical staff to provide preoperative explanations, post-operative structured counselling (16), and partner support or mutual-help groups (17). However, such medical resources are severely lacking in remote and underdeveloped areas. Some patients refuse post-operative follow-up due to financial constraints, resulting in their inability to accept structured psychological counselling. Current intervention methods require advanced planning and are conducted at fixed times and locations. However, the emotional distress is often sudden and unpredictable, making it difficult for traditional offline interventions to provide immediate emotional support, which may exacerbate the condition. Developing online intervention methods can compensate for the aforementioned shortcomings, but research in this field remains largely unexplored. Wang et al. developed an online short-term intervention for patients who had undergone abortions based on stress coping theory, which improved psychological well-being by enhancing self-efficacy and social support. The results showed that two weeks after the abortion, the intervention group had significantly lower depression scores, improved problem-coping abilities, and held more positive perceptions of their abortion experiences (18).

Mobile health is “medical and public health practices supported by mobile devices, such as mobile phones, patient monitoring devices, personal digital assistants (PDAs), and other wireless devices.” (19) The application of mobile health in medicine is becoming increasingly widespread. Its main advantages are that it can break through the limitations of time and space, is inexpensive to use, and can provide fast, rich, and personalized medical information (20).

Large language models (LLMs) are complex neural network models based on deep learning, specifically designed to understand, process, and generate human-like natural language. They are trained on massive amounts of text data, learning statistical patterns and contextual relationships within the text to accomplish tasks (21). At present, commonly used LLMs internationally include ChatGPT, etc. Recently, LLMs in China have gradually developed, and models such as DeepSeek, Doubao, and Kimi have also gained recognition and widespread application in China. The application of LLMs in medicine includes simplifying complex medical information, integrating patient data, assisting doctors in clinical diagnosis and decision-making, and performing common medical and health tasks such as health education and emotional support for patients through the generation of high-quality, human-like interactive text (22).

LLMs are primarily deployed on Personal Computers (PCs) and some mobile devices, with deployment methods depending on model size, optimization level, and mobile device hardware capabilities (23). Successfully deploying a fully functional LLM on mobile phones can combine its exceptional natural language processing capabilities with the advantages of mobile health, which breaks through spatial and temporal limitations.

Coze is a new-generation AI agent development platform developed by ByteDance, enabling the rapid creation of various AI applications based on LLMs. With its modular workflow engine and robust knowledge base construction capabilities, it effectively mitigates the phenomena of “hallucination” or “fabrication” in LLMs (24), ensuring the accuracy of responses in professional domains. The platform supports users uploading their databases, allowing agents to complete self-learning based on this data. Through its one-click deployment function, AI agents can be deployed to WeChat Official Accounts, leveraging WeChat’s over 1.4 billion active users to provide precise services for professional settings.

Swanson’s Theory of Caring was developed by British scholar Swanson, whose theoretical framework integrates the core findings of three independent phenomenological studies. Swanson’s Theory of Caring emphasizes that nurses are the primary implementers of patient care, possessing a high degree of independence and not merely serving as assistants to doctors. The theory posits that nurses’ care and concern for patients have a positive impact on patient health that is no less significant than the therapeutic interventions. Its core framework consists of five care behaviors: knowing, being with, doing for, enabling, and maintaining belief (25).

1. Knowing: Nurses assess patients’ needs and physical and psychological status thoroughly. Knowing patients well is the basis for personalized care. But be careful not to be prejudiced. Use a variety of comprehensive assessment methods and focus on patients’ concerns and confusion.
2. Being with: Being with someone is a step beyond knowing them. Nurses in clinical settings focus on emotional support, which can be done by being physically present with patients, encouraging them to express their feelings, sharing their own experiences and feelings with patients, and responding appropriately. This helps patients feel cared for, supported, and understood.
3. Doing for: This typically refers to nurses providing professional care to patients and offering practical assistance, such as pain management and technical guidance. However, excessive intervention is not recommended.
4. Enabling: Nurses need to use their professional knowledge to provide patients with information and explanations through education and resource provisioning, offer emotional support, and enhance patients’ self-care abilities.
5. Maintaining belief: Nurses need to help patients maintain hope, enable them to get through events or turning points, and face the future positively.

Currently, Swanson’s Theory of Caring is widely applied in studies involving women who have experienced abortion. Jansson et al. (26) conducted semi-structured interviews with women who experienced early abortion or missed abortion between 18 and 20 weeks of pregnancy. The researcher analyzed the data using the five processes of Swanson’s Theory of Caring. The results indicated that care beneficial to women includes empathy, emotional support, maintaining dignity, competence, and meeting individualized needs. Adolfsson et al. (27) applied Swanson’s Theory of Caring to the rehabilitation of women who experienced early miscarriage. The results indicated that midwives who followed up with women after miscarriage using this theory as a guide were able to reduce sadness scores. Palas et al. (28) found that personalized care based on Swanson’s Theory of Caring could alleviate patients’ physical, emotional, behavioral, and cognitive sadness symptoms, as well as reduce future negative emotions, depression, and anxiety.

The study aims to evaluate the effectiveness of an Artificial Intelligence-Empowered Mobile Health intervention using Swanson’s Theory of Caring on negative psychological states such as depression and anxiety in women after abortion through a mixed-methods study, while also examining clinical examination and test indicators and semi-structured interview data from patients.

The specific objectives are:

1. To evaluate whether the two-week AI mobile health intervention program reduces psychological distress in women after miscarriage.
2. To evaluate the acceptability of an AI-based mobile health intervention in primary care for women after abortion.

## 2 Materials and methods

### 2.1 Design

**Figure 1:**
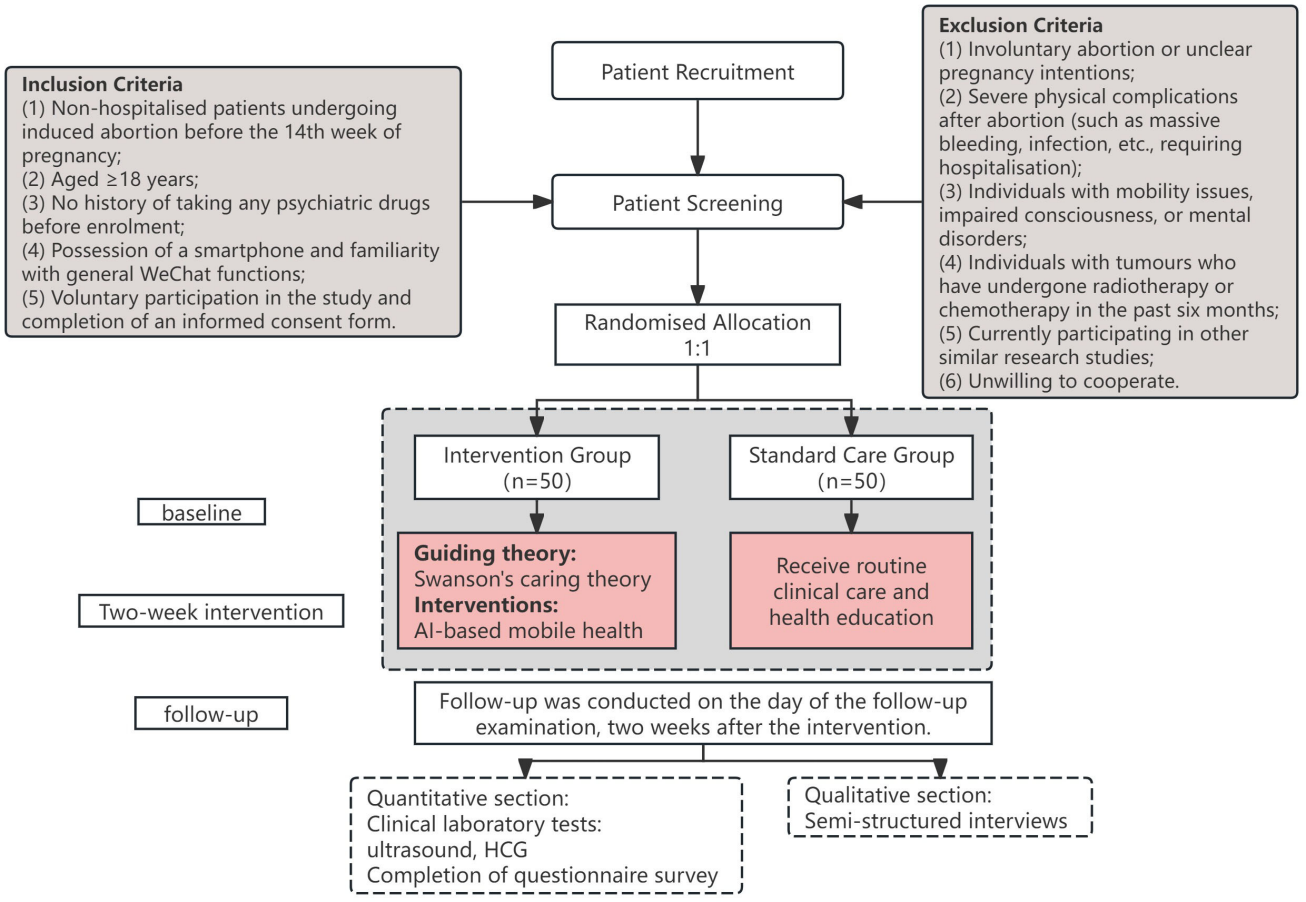
Flow chart of patient recruitment and study implementation.

The study will use a mixed-methods study design and will be conducted at the Inner Mongolia Maternity and Child Health Care Hospital from 2025 to 2026. A randomized controlled trial with two parallel groups will compare the effectiveness of an Artificial Intelligence-Empowered Mobile Health intervention guided by Swanson’s Theory of Caring on psychological distress (such as depression, anxiety, and stress) in women after abortion. The intervention will last for two weeks, with participants undergoing questionnaires, physical examinations, and individual interviews at baseline and at two weeks to explore changes in psychological distress. The study flowchart is shown in **Figure 1**.

The study used a simple randomization method. The steps are as follows: first, in Excel, we used the RAND function to generate a number between 0 and 1. The function is invoked by entering “=RAND()” in any cell and pressing Enter. The generated number determines the group assignment for each patient: if the number is from 0 to 0.5 (excluding 0.5), the patient is assigned to the intervention group; if it is from 0.5 to 1 (excluding 0.5), the patient is assigned to the control group. If the number is exactly 0.5, the process is repeated until a valid random number is obtained.

The blinded method used in this study involves several categories of individuals:

1. Medical staff in the research department (blinded): responsible for patient recruitment, informing patients of admission and follow-up times and locations, and conducting basic biochemical and imaging examinations for admission and follow-up, they are unaware of patient group assignments.
2. Investigators (blind): Their responsibilities are strictly limited to one-on-one data collection with patients; they do not participate in other research activities to ensure data specificity and accuracy. Investigators are unaware of patient group assignments.
3. Data processing personnel (blind): They receive coded data (Group A/Group B) and are unaware of the specific assignment relationship between Groups A/B and the intervention or control groups.
4. Patients (unblinded): The intervention group is guided by the researcher to follow the Artificial Intelligence-Empowered Mobile Health Official Account and instructed on its use. The control group does not receive this service.
5. System administrator (unblinded): Responsible for online management and answering questions for patients in the intervention group; their service is limited to patients in the intervention group, as only this group receives the Artificial Intelligence-Empowered Mobile Health intervention.

### 2.2 Study sample

A total of 100 participants will be enrolled in this study. The participants in this study were female outpatients who undertook surgical abortion at the Inner Mongolia Maternity and Child Health Care Hospital. This study used continuous enrolment, and prior to the study, patient consent was obtained, and patients were randomly assigned to the intervention group and the control group, with 50 patients in each group, for a total of 100 patients. The inclusion and exclusion criteria are as follows:

#### 2.2.1 Inclusion criteria

1. Non-hospitalized patients undergoing surgical abortion before the 14th week of pregnancy;
2. Aged ≥18 years;
3. No history of taking any psychiatric drugs before enrolment;
4. Possession of a smartphone and familiarity with general WeChat functions;
5. Voluntary participation in the study and completion of an informed consent form.

#### 2.2.2 Exclusion criteria

1. Involuntary abortion or unclear pregnancy intentions;
2. Severe physical complications after abortion (such as massive bleeding, infection, etc., requiring hospitalization);
3. Individuals with mobility issues, impaired consciousness, or mental disorders;
4. Individuals with tumors who have undergone radiotherapy or chemotherapy in the past six months;
5. Inability to understand or complete questionnaires in Chinese;
6. Currently participating in other similar research studies;
7. Unwilling to cooperate.

### 2.3 Sample size

This study is a mixed-methods research. The intervention group will receive Artificial Intelligence-Empowered Mobile Health interventions, while the control group will receive standard care. Based on previous research findings, nursing interventions based on Swanson’s Theory of Caring have been shown to significantly reduce depression and anxiety levels in women who have experienced abortion, and can serve as a reference for effect size (29). The Minimum Clinically Important Difference (MCID) was selected as a reduction of ≥5 points on the Patient Health Questionnaire-9 (PHQ-9), which is considered clinically meaningful (30). The standard deviation (SD) is based on previous studies showing that the SD of PHQ-9 in the abortion population is approximately 6–8 points. The test level α = 0.05 and test efficacy 1-β = 0.90 are set, and according to the randomized controlled trial mean comparison formula:


$$n=\frac{{\left(Z_{\alpha /2}+Z_{\beta }\right)}^{2}\cdot 2\sigma^{2}}{\Delta^{2}}$$

The sample sizes for each experimental group are equal. Z_α/2_ = 1.96, Z_β_ = 1.28, σ = 8, Δ = 5 in this example. The sample size for each group can be calculated as n = 42 people. Considering the follow-up missing rate, the sample size for each group is increased to 50 people, for a total of 100 people.

Qualitative research is guided by the principle of information saturation, meaning that interviews with patients are stopped once a certain number of participants have been reached and no new information is emerging. Therefore, this study does not specify a minimum sample size for qualitative research.

### 2.4 Recruitment

#### 2.4.1 Quantitative research section

1. One month prior to the formal survey, recruitment will be promoted through posters within the hospital and other means, and medical staff from relevant departments will contact potential research participants via telephone, face-to-face interviews, or online communication.
2. One day prior to the formal survey, research participants will be contacted to briefly explain the research content and confirm the baseline enrolment time.
3. On the day of baseline enrollment, explain the purpose, methods, and follow-up arrangements of the study to the participants, commit to anonymizing the questionnaire data to protect privacy, and obtain informed consent. Explain that the service account usage fees will be covered by the project, and that participants will receive a gift each time they participate in the survey.

#### 2.4.2 Qualitative research section

1. In the quantitative research, two categories of patients were selected based on their baseline PHQ-9 scores: those with higher scores and those with lower scores. The selection process ensured representativeness across key demographic characteristics such as gender and age, resulting in an initial list of interview participants.
2. Prior to the formal interviews, patients were contacted through multiple ways to explain the content of the qualitative research and obtain informed consent, and interview times were scheduled.
3. On the day of the interview, the research objectives, methods, and recording permissions and privacy protection measures were reiterated to the participants. After obtaining consent, the interview was initiated, and a participation gift was provided at the end.

### 2.5 Interventions

#### 2.5.1 Control group: standard care

The control group was not completely blank. At the time of enrolment, patients in the control group received standard oral health education, including explanations of the surgical procedure and precautions, routine preoperative examinations, and guidance on postoperative precautions. Any questions or concerns raised by patients were answered in detail.

#### 2.5.2 Intervention group: standard care + artificial intelligence-empowered mobile health

Based on the physiological recovery cycle after abortion and the critical time window for screening complications, this study was designed as a two-week continuous intervention. The intervention protocol was developed by a multidisciplinary team comprising public health experts, clinician doctors, nurses, psychologists, and statistical analysts, integrating expertise from all relevant fields. The core intervention method employs Artificial Intelligence-Empowered Mobile Health, guided by Swanson’s Theory of Caring as a theoretical framework. The two-week intervention period is divided into distinct phases, each of which is strictly implemented by the fundamental care dimensions of this theory.

#### 2.5.3 Preparation before intervention

To overcome the “hallucination” and “fabrication” problem of LLMs, this study formed a multidisciplinary team of medical professionals to build a structured Knowledge-based Question Answering (KBQA) and put it into use through an FAQ robot. The team comprised specialists from multiple medical disciplines, including two obstetricians and gynecologists, three registered nurses with over five years’ experience in obstetrics and gynecology, a clinical psychologist, and a public health specialist. The research team will randomly sample approximately 5% of AI-patient interaction logs daily for review. Based on the five core processes of Swanson’s theory (Knowing, Being with, Doing for, Enabling, Maintaining belief), the theoretical adherence of interaction content will be assessed. Should any deviation be identified, the team will immediately convene to revise knowledge base content, ensuring the sustained quality and theoretical consistency of the intervention.

The knowledge base covers core content such as medical knowledge about abortion, post-operative diet, and exercise guidance etc.(The daily checklist is detailed in the Supplement1). The knowledge base is deployed on the Coze, where the LLMs can self-learn and provide 24-hour professional Q&A services via a WeChat Official Account. After each query, the system recommends three related questions to guide patients into deeper interaction. Researchers regularly analyze interaction data to identify patients’ concerns and severe physiological and psychological symptoms, contact attending physicians when necessary, and continuously update the knowledge base according to the quality of Q&A and user needs.

After conducting multiple model comparisons and adjustments, the “Doubao-pro-32k” was ultimately selected for use in this study. The “Doubao-pro-32k” model is an advanced large language model (LLM) provided by the Coze platform, specifically the version identified as Doubao-1.5-thinking-pro/250415. It is a text-only model that excels in specialized domains such as mathematics, programming, and scientific reasoning, as well as general tasks like creative writing. The model has demonstrated competitive performance on several authoritative benchmarks, including AIME 2024, Codeforces, and GPQA, achieving results that are comparable to or near the top tier of current industry standards.

This decision was based on research indicating that Doubao-pro-32k achieves a higher overall score and demonstrates outstanding performance in knowledge application among similar LLMs used in medical applications (31). After the initial development is completed, the experts’ department of obstetrics and gynecology will be invited to evaluate the quality of the LLM’s responses to ensure accuracy and professionalism. Additionally, the WeChat Official Accounts only access the user’s WeChat nickname, avatar, signature, and location; the account does not involve any related privacy data.

#### 2.5.4 Specific intervention strategies

**Phase 1: From outpatient visit to surgery day.**

**Swanson’s Theory of Caring:** Knowing.

**Intervention providers:** Attending doctor, researcher.

**Intervention content:**

1. Patients fully understand the intervention process and measures of the study.
2. Doctors and researchers fully understand the patient’s medical history and psychological needs.

**Specific Methods:**

1. Study Introduction and Trust Building: During outpatient visits, the researcher and attending doctors jointly provide patients who meet the inclusion criteria and are willing to participate in the study with a comprehensive introduction to the study, thereby establishing an initial trust relationship with the patients.
2. Establishment of Patient Information Records: For patients who meet the study criteria and are included in the study, the researcher guides them in completing electronic questionnaires via Questionnaire Star after signing the informed consent form. Basic medical conditions and laboratory results are recorded in the hospital’s medical record system.
3. In-depth Interview Research: Researchers engage in in-depth communication with patients, asking questions such as “What do you think is the biggest problem you are facing right now?” and “What are your strongest feelings about abortion?” to gain a detailed understanding of the patients’ thoughts and needs.

**Swanson’s Theory of Caring:** Doing for, Enabling.

**Intervention providers:** Researcher.

**Intervention content:**

Provide patients with detailed information about the functions of Artificial Intelligence-Empowered Mobile Health, demonstrate its operation, and guide AI questioning.

**Specific Methods:**

1. Detailed Description of Functions:

Artificial Intelligence-Empowered Mobile Health provides the following support:

① Health Education: Answers questions about diseases, such as preoperative preparation, potential complications, recovery time, etc., guides physical recovery, such as the normal range of bleeding days and amounts, indications for medical treatment, etc., provides dietary advice, such as dietary taboos, common dietary misconceptions, etc., provides exercise guidance, such as bed rest time, appropriate types of exercise and timing, etc.

② Entertainment and Emotional Support: Artificial Intelligence-Empowered Mobile Health has personification attributes, can introduce itself during the first interaction, can recognize user emotions and provide comfort and encouragement, and also provides practical tools such as weather queries and simple calculations.

2. Artificial Intelligence-Empowered Mobile Health Usage Process Guidance:

Step 1: Researchers provide one-on-one guidance to patients who meet the criteria and have signed the informed consent form, instructing them to follow the WeChat Official Account for this study on their mobile phones.

Step 2: Guide patients to enter the Official Account and locate the chat interaction page.

Step 3: Under the guidance of the researcher, patients take the lead in operating the Official Account, using text or voice input to experience its health education and emotional support functions.

3. Question Guidance:

Researcher demonstrated common input commands face-to-face, such as “What do I need to prepare for an abortion?” and guided patients on how to ask questions effectively to the Official Account. The researcher provided concise answers to other questions from patients about the system’s functions and operation, and performed live demonstrations of the functions on the patients’ mobile phones.

**Swanson’s Theory of Caring:** Being with, Maintaining belief.

**Intervention providers:** Attending doctor, researcher.

**Intervention content:**

1. Emotional Presence: Introduce relevant personnel and treatment processes, assist families in need, and convey a sense of companionship.
2. Give Courage: Share cases to alleviate patients’ fears.

**Specific Methods:**

1. Staff Introduction: Introduce the attending doctor and nurse during the first consultation to establish comfort and familiarity.
2. Detailed Explanation: Provide an everyday language explanation of the purpose of the examination and important considerations. For example, a blood routine is conducted to exclude anemia and ensure safety, and an ultrasound requires holding urine and drinking water beforehand. Visualize medical reports, such as describing “a gestational sac size of 5 cm” as “the size of an egg, consistent with the gestational age.”
3. Body Language: Use eye contact, physical touch, and accompany patients during waiting periods to alleviate anxiety and feelings of loneliness.
4. Provide Support Tailored: Explain medical insurance policies and exemption procedures in person for economic difficulties, provide dialect interpretations (with QR codes) and handwritten versions of important notes for those with lower educational levels.
5. Empowerment Through Similar Cases: Provide successful case examples with similar clinical and social backgrounds to enhance patients’ confidence and courage in recovery.

**Phase 2: Two weeks after surgery and before follow-up examination**.

**Swanson’s Theory of Caring:** Knowing.

**Intervention providers:** Attending doctor, researcher.

**Intervention content:**

Assess postoperative complications, physical recovery status, and current psychological state.

**Specific Methods:**

1. Communication Time: One week after the patient’s abortion, online communication via telephone, WeChat, or other platforms, lasting approximately 20 minutes.
2. Communication Content: Comprehensive understanding of the patient’s physical recovery, inquiring about fever, foul-smelling secretions, bleeding, abdominal pain, etc., assessing the patient’s psychological state, whether there are negative emotions such as inferiority, anxiety, depression, or despair, recognizing the patient’s additional needs for post-operative health knowledge.

**Swanson’s Theory of Caring:** Doing for, Enabling.

**Intervention providers:** Researcher.

**Intervention content:**

1. Send brief post-operative instructions via WeChat to guide patients in using Artificial Intelligence-Empowered Mobile Health.
2. Monitor data in the system and provide feedback updates.

**Specific Methods:**

1. Check the Official Account function before 8:00 every day to ensure that the AI response is working properly.
2. Send brief post-operative guidance and education via WeChat at 9:00 every day, and guide consultations through questions about common misconceptions. For example: “Is it true that you can’t eat fish after an abortion?” Please consult “Xiao Yu” (the name of the Artificial Intelligence-Empowered Mobile Health agent) for an authoritative answer!
3. Review patient-AI interaction records by 9:00 PM daily, summarize key feedback for the attending doctor, and simultaneously update the knowledge base with missing content.

**Swanson’s Theory of Caring:** Being with, Maintaining belief.

**Intervention providers:** Researcher.

**Intervention content:**

1. Guide patients to express their emotions appropriately.
2. Encourage patients to develop hobbies and interests.
3. Encourage family members to accompany patients.
4. Inform patients of follow-up examination arrangements and precautions.

**Specific Methods:**

1. Artificial Intelligence-Empowered Mobile Health provides patients who lack emotional outlets with humanized emotional release and real-time companionship support, filling the gap left by their reluctance to express themselves or insufficient family support.
2. Artificial Intelligence-Empowered Mobile Health can provide recommendations for activities of interest and help patients plan their schedules, helping them avoid lying in bed alone, alleviating negative emotions, and solving their difficulties in choosing activities.
3. Artificial Intelligence-Empowered Mobile Health solutions use human-like imagery and interactive entertainment features to provide alternative emotional support for patients living alone or lacking family support.
4. Strengthen the connection between patients and researchers to identify serious issues and initiate timely medical interventions. Inform patients of follow-up appointment times, locations, procedures, required materials, and preparation requirements, such as fasting or holding urine.

**Phase 3: Follow-up to ongoing treatment**.

**Swanson’s Theory of Caring:** Knowing.

**Intervention providers:** Attending doctor, researcher.

**Intervention content:**

1. Conduct clinical examinations to assess recovery.
2. Collect patients’ opinions on the study.

**Specific Methods:**

1. Conduct inquisition, physical examinations, and necessary auxiliary examinations, and promptly provide feedback and explanations of the results to patients.
2. Collect patient responses and impressions regarding the use of Artificial Intelligence-Empowered Mobile Health through semi-structured interviews, and guide completion of follow-up questionnaires.

**Swanson’s Theory of Caring:** Doing for, Enabling.

**Intervention providers:** Researcher.

**Intervention content:**

1. Artificial Intelligence-Empowered Mobile Health provides patients with a continuous interaction period of three months.
2. Artificial Intelligence-Empowered Mobile Health offers long-term care knowledge after abortion.

**Specific Methods:**

1. On the day of the follow-up examination, the doctor will provide face-to-face guidance on key points for long-term post-operative care.
2. Patients will be instructed that they can consult on authoritative social media platforms if they encounter any issues.

**Swanson’s Theory of Caring:** Being with, Maintaining belief.

**Intervention providers:** Researcher.

**Intervention content:**

Researchers provide accompaniment and guidance to patients during follow-up appointments.

**Specific Methods:**

1. Contact the patient proactively before the follow-up examination to reconfirm the time, location, and examination items.
2. Accompany the patient throughout the follow-up examination on the day of the appointment, explaining the environment and the purpose of each examination to alleviate any feelings of unfamiliarity.
3. Pay attention to the patient’s emotions, provide patient companionship and positive support, and encourage them to face the future with optimism.

### 2.6 Outcomes

#### 2.6.1 Primary outcome

##### 2.6.1.1 Depression levels

In this study, symptoms of depression were assessed using PHQ-9. This scale consists of nine items, each corresponding to the core symptoms of depression in the Diagnostic and Statistical Manual of Mental Disorders (DSM), such as depressed mood, sleep problems, and fatigue (30). Each item is scored on a scale of 0–3 points(0 = “not at all” to 3 = “nearly every day”). The scores for the 9 items are added together, with a total score ranging from 0–27 points. The higher the total score, the more severe the depressive symptoms. The severity of depression is classified according to the total score as follows: 0–4 minimal depression, 5–9 mild depression, 10–14 moderate depression, 15–19 moderate depression, 20–27 severe depression. The Cronbach’s alpha for the Chinese version of the PHQ-9 is 0.86 (32).

#### 2.6.2 Secondary outcomes

##### 2.6.2.1 Psychological and behavioral health

Anxiety symptoms are measured using the three-item short form of Generalized Anxiety Disorder (GAD-3). Each item is scored on a scale of 0–3 points(0 = “not at all” to 3 = “nearly every day”). The scores for the three items are added together, with a total score range of 0–9 points. With higher scores indicating greater anxiety levels, the RiskSLIM algorithm assigns different weights to each item to form a weighted total score. The optimal practical cutoff value for GAD-3 is 15 (33). The Cronbach’s α for this scale among nursing students was 0.934 (34).

Stress levels were measured using the Perceived Stress Scale-4 (PSS-4). It consists of four items, and each item is scored on a scale of 0–4 points(0 = “never” to 4 = “very often”), including two reverse-scored items. The total score ranges from 0 to 16 points, with higher scores indicating more significant psychological stress in patients (35, 36). The Chinese version of the PSS-4 demonstrated a Cronbach’s alpha of 0.473 when validated among a large community-based general population. In a study examining the relationship between perceived stress and marital satisfaction among infertile couples, the Cronbach’s alpha coefficient for the PSS-4 was 0.572 (37).

Social support is measured using the Perceived Social Support Scale Short Form (PSSS-SF). This scale consists of three items designed to assess an individual’s self-perceived levels of social support. Each item is scored on a scale of 1 to 7 points (1 = “not at all compatible” to 7 = “fully compatible”). The total score range for this scale is 3 to 21, with higher scores indicating higher levels of perceived social support (38).

The Body Image–Acceptance and Action Questionnaire–5 (BI-AAQ-5) is used to measure body image flexibility. The scale consists of 5 items. Each item is scored on a scale of 1 to 5 (1 = “not at all compatible” to 5 = “fully compatible”). Some items require reverse scoring. The total score range for the scale is 5 to 25 points. Higher scores indicate greater acceptance of body image and fewer avoidance behaviors toward negative body experiences. The overall Cronbach’s alpha for the scale during development was 0.85 (39, 40).

The Posttraumatic Stress Disorder (PTSD) Checklist for DSM-5 (PCL-5) is used to measure symptoms that meet the criteria of the DSM-5. The scale consists of four dimensions: intrusive symptoms, avoidance, cognitive/emotional negative changes, and hyperarousal, with a total of 20 items. Each item is scored on a scale of 0 to 4 (0 = “not at all” to 4 = “extremely “). The total score range is 0–80 points, with higher scores indicating more severe PTSD symptoms and greater functional impairment. The overall Cronbach’s alpha for the original scale was 0.94 (41).

Quality of life is measured using the EuroQol Visual Analogue Scale (EQ-VAS), which is used to assess the overall health status of participants. The EQ-VAS is a vertical scale on which respondents record their self-rated health status (42). The scale ranges from 0 to 100, with 0 representing “the worst health state you can imagine” and 100 representing “the best health state you can imagine”.

Physiological and biochemical data: Professional medical staff at the Inner Mongolia Maternity and Child Health Care Hospital, who are qualified to perform such procedures, strictly adhere to clinical operating procedures and hospital testing standards to conduct human chorionic gonadotropin (HCG) level measurements and ultrasound examinations on patients. The HCG level measurement employs chemiluminescence immunoassay, utilizing the advanced, fully automated chemiluminescence immunoassay analyzer to precisely quantify the level of human chorionic gonadotropin in serum. Ultrasound examinations are conducted using a color Doppler ultrasound diagnostic instrument, with experienced ultrasound specialists conducting detailed examinations and assessments of uterine morphology, endometrial thickness, and the presence of any residual material within the uterine cavity.

Qualitative interview: Researchers initially developed a semi-structured interview outline based on the research objectives and a review of relevant literature. After consulting with qualitative research experts and conducting preliminary interviews, the research team revised the interview outline based on expert discussions and finalized the content of the interview outline. During the interviews, researchers needed to maintain a certain degree of openness and flexibility. The outline included, but was not limited to, the following questions:

1. What specific functionalities of Artificial Intelligence-Empowered Mobile Health did you use?
2. Which Artificial Intelligence-Empowered Mobile Health functionalities had a positive effect on your health, and which ones had no effect or even had a negative effect?
3. How did using Artificial Intelligence-Empowered Mobile Health affect your mental health? What do you think caused these effects?
4. With the help of Artificial Intelligence-Empowered Mobile Health, what did you learn about post-abortion care?
5. To what extent did you trust the health information provided by Artificial Intelligence-Empowered Mobile Health? Did you find its responses reliable? Why?’
6. How did you feel when Artificial Intelligence-Empowered Mobile Health attempted to comfort or encourage you? Do you perceive this emotional support as genuine? Please elaborate on your feelings.”
7. What were your expectations of Artificial Intelligence-Empowered Mobile Health prior to using it? How did its actual performance differ from your expectations?’
8. Compared to support provided by healthcare professionals or family/friends, what unique aspects did you find in interacting with Artificial Intelligence-Empowered Mobile Health? Which form of support do you prefer? Why?’

Patient demographic information will be collected through the case system and self-designed questionnaires. (**Table 1**).

**Table 1:** Variables and measures collected at every time point.

Variables	Date source	Measures	Baseline(T0)	Post-intervention(T1)
Intervention preparation	/	Eligibility screen, Informed consent and Allocation	✓	
Covariates	Baseline questionnaire	Sociodemographic information	✓	
	Baseline questionnaire and Follow-up questionnaire	Behavioral information	✓	✓
Primary outcome	Baseline questionnaire and Follow-up questionnaire	Patient Health Questionnaire-9 (PHQ-9)	✓	✓
Secondary outcome	Baseline questionnaire and Follow-up questionnaire	Generalized Anxiety Disorder-3, GAD-3	✓	✓
		Perceived Stress Scale 4, PSS-4	✓	✓
		Perceived Social Support Scale Short Form, PSSS-SF	✓	✓
		Body Image-Acceptance and Action Questionnaire-5, BI-AAQ-5	✓	✓
		Posttraumatic Stress Disorder Checklist for DSM-5, PCL-5	✓	✓
		EuroQol Visual Analogue Scale, EQ-VAS	✓	✓
	Clinical examination and laboratory tests	Ultrasound, human chorionic gonadotrophin, HCG		✓
Qualitative results	semi-structured interviews	Effect of the trial		✓

### 2.7 Statistical analysis

After the trial is completed, all collected case data and questionnaire responses will be organized and summarized. Data storage will be managed using Microsoft Office 2017, while statistical analysis will be conducted using SPSS Statistics 27.0 software. Count data will be presented as case numbers (N) and percentages (%). The normality of continuous data will be assessed; normally distributed data will be presented as mean ± standard deviation, while non-normally distributed data will be presented as median (interquartile range) and interquartile range (P25-P75). Appropriate statistical tests (e.g., independent samples t-test, Mann-Whitney U test, and chi-square test) will be used to compare baseline characteristics between the intervention and control groups based on data type. Longitudinal analysis of follow-up data will be conducted using generalized estimating equations (GEE) to compare differences in outcomes between the two groups. Two-sided tests will be used, with statistical significance set at p < 0.05. In cases of missing data, multiple imputation techniques will be applied, considering the randomness of missing data. Multiple imputation involves generating multiple complete datasets by replacing missing values with reasonable estimates derived from observed data and imputation models. Compared to single imputation methods, this approach preserves the variability of the dataset and enhances the reliability of results. Multiple imputation will be performed using established statistical software (e.g., SPSS or R). Sensitivity analysis will also be conducted to assess the robustness of results under various assumptions regarding missing data. This comprehensive approach ensures the validity and reliability of research findings while maintaining statistical integrity throughout the analysis process and accounting for missing data. Qualitative interview audio files will be transcribed verbatim and anonymized, and analyzed using Grounded Theory with NVivo (Version 12, QRS International, Doncaster, Australia). Qualitative and quantitative data will be incorporated to better interpret the results across multiple stages during the trial.

### 2.8 Ensuring intervention compliance

To encourage subject compliance, we will (1) fully explain the subject’s responsibilities during the observation period at the time of recruitment and signing of the informed consent form. At the same time, we will strive to build strong relationships with participants with the aim of promoting mutual trust. (2) Throughout the study, researchers will provide short- and long-term self-care guidance and comprehensive health education after abortion. (3) The research team will assess the interaction between the intervention group and the system on a weekly basis. This is to ensure that participants are fully engaged. Regular monitoring can identify any issues or dropouts promptly, enabling the team to take corrective actions and maintain participant engagement.

### 2.9 Study management

All research data (questionnaires, baseline medical conditions, and examination results) were de-identified upon collection and entered into a secure institutional server maintained by the Information Department of Inner Mongolia Maternity and Child Health Care Hospital. Data are stored within a controlled-access partition equipped with firewalls and intrusion detection systems. Access to the data is restricted to the Principal Investigator (PI) of this study and authorized statistical personnel, who utilize personal encrypted credentials. Data storage and backup procedures adhere to the hospital’s electronic information security policy. All data will be retained for a minimum of ten years following the conclusion of the research project, after which it will be securely destroyed.

The Data Monitoring Committee (DMC) consists of at least two members of the Ethics Committee of the Inner Mongolia Maternal and Child Health Hospital, who have no conflict of interest with the study. The DMC is responsible for conducting regular monitoring independently of the principal investigator and reporting to the Ethics Committee. It has the authority to suspend or terminate any study that deviates from the protocol. The Ethics Committee reviews the progress of the study and compliance with the protocol every six months. The research team must immediately report any serious adverse events (SAEs) or adverse events (AEs). If a participant experiences a serious adverse event, researchers must report it within 24 hours and halt the trial. In the event of a severe error in the intelligent agent or significant interaction issues, the trial must also be immediately suspended. The study may resume only after technical repairs and interface improvements have been confirmed. A pre-trial validation of the intelligent agent’s performance is conducted prior to study initiation, and all investigators undergo rigorous training and certification. In a standardized environment, investigators guide participants one-on-one to scan a QR code and complete a unified electronic questionnaire, explaining questions without prompting answers. Prior to the interview, participants are fully informed of the purpose, right to withdraw, and confidentiality measures. After obtaining signed informed consent, the interview is recorded and conducted in a private setting at the pre-agreed time. During the interview, investigators flexibly guide the conversation, listen attentively, and avoid subjective evaluations. In this study, we will utilize the “Wenjuanxing” (Questionnaire Star) online survey platform for questionnaire completion to ensure data accuracy. Before data analysis, we will conduct strict data organization and cleaning.

## 3 Discussion

Abortion places multiple physical, psychological, and economic burdens on women. Physical complications can be effectively monitored and mitigated through hospital observation and regular follow-up examinations; however, there is a serious lack of systematic intervention for post-operative mental health. The current practice of relying primarily on pre-operative or follow-up consultations with medical staff has significant limitations: (1) there is a lack of continuous support throughout the post-operative period; (2) medical staff have limited time for in-depth communication (43); (3) Trust between patients and healthcare providers has not been established during the initial consultation, which may lead to resistance (44); (4) The offline model is constrained by time and space, making it difficult to respond promptly to emotional crises that arise outside of working hours (e.g., late at night).

This study addresses existing limitations by providing intervention throughout the entire process of abortion treatment using Artificial Intelligence-Empowered Mobile Health under the guidance of Swanson’s Theory of Caring, intending to reduce the incidence of negative emotions in patients. Its innovation lies in the first application of Artificial Intelligence-Empowered Mobile Health using LLM to women undergoing abortion: through systematic screening, multiple rounds of expert consultation, and workflow testing, a timely, accurate, and highly specialized intelligent agent was constructed. Guided education promotes patient use of Artificial Intelligence-Empowered Mobile Health, builds trust, and enables real-time interaction, breaking through the spatial and temporal limitations of traditional interventions. The low-cost attribute of Artificial Intelligence-Empowered Mobile Health alleviates patients’ economic concerns. By integrating authoritative literature and expert consensus, Artificial Intelligence-Empowered Mobile Health helps mitigate regional disparities in medical resources, paving new pathways for promoting health equity (45, 46).

Although the intervention measures in this study have certain potential, the study has several limitations. Single-center sampling relies on voluntary participation, which may underestimate the representativeness of groups with low health literacy, technological disadvantages, and stigma, leading to selection bias. The inclusion criterion may have inadvertently and systematically excluded female groups with lower socioeconomic status, limited digital literacy, or residing in remote areas with poor internet coverage. This exclusion not only renders the research sample insufficiently representative of these groups, thereby introducing selection bias, but also limits the external validity of findings to women possessing certain technological and resource advantages. Consequently, the experiences and needs of resource-deprived, vulnerable women who genuinely require support remain inadequately captured and reflected. Consequently, we shall cautiously define the applicability of our findings, clarifying that they primarily pertain to populations with comparable technological access and capabilities. Future research should explore offline or low-tech intervention models to bridge the digital divide and extend benefits to these excluded vulnerable groups. This represents a crucial direction for advancing equity in post-abortion mental health services for women.
