# Supplementary material for: The effect of artificial intelligence-empowered mobile health on psychological distress in women following abortion: protocol for a mixed-methods study
Source: Front Psychiatry. 2025 Nov 18;16:1665500. doi: 10.3389/fpsyt.2025.1665500 (PMC12746660; doi:10.3389/fpsyt.2025.1665500)
Supplement: Supplementary file 1 [file Table1.docx]

Supplement1

**Artificial Intelligence-Empowered Mobile Health Intervention Daily Checklist**

Intervention Period: Postoperative Day 1 to Day 14

Core Platform: WeChat Official Account (AI agent named ‘Xiao Yu’)

Guiding Theory: Swanson's Caring Theory (Knowing, Being with, Doing for, Enabling, Maintaining belief)

Delivery Time: Daily at 09:00

Implementer: Researcher

Daily Fixed Procedure:

08:00: Verify functionality of ‘Xiao Yu’ AI service account.

09:00: Dispatch daily scripted messages to intervention group patients via WeChat.

Before 21:00: Review patient interactions with ‘Xiao Yu’, summarise key information (e.g., prevalent queries, severe emotional concerns) for feedback to attending physicians, and update knowledge base accordingly.

| Week | Day | Swanson's Theory of Caring Dimension | Educational Script  (Concise & Easy-to-Understand) | Guiding Question Script  (To guide patients to use the "Xiao Yu" AI) |
| --- | --- | --- | --- | --- |
| **Week 1** | **Day 1** | **Knowing** | Light bleeding and mild abdominal pain after abortion are normal and usually last 1-2 weeks. If bleeding exceeds your menstrual flow or you have a fever, please contact us promptly. | How are you feeling today? You can ask "Xiao Yu" about normal bleeding volume and recovery signs anytime! For example: "Xiao Yu, what is considered normal bleeding?" |
| **Week 1** | **Day 2** | **Being with** | Emotional fluctuations after the procedure are very normal. Please allow yourself to have these feelings without excessive worry. We and "Xiao Yu" are here with you. | How is your mood today? If you need someone to talk to, "Xiao Yu" is here to chat. It will be a patient listener. |
| **Week 1** | **Day 3** | **Doing for** | Your body needs nutrition to recover. We recommend eating foods rich in protein and iron, like eggs, lean meat, and leafy green vegetables. Avoid raw, cold, and spicy foods. | Have you planned your meals for today? If you're unsure what to eat, ask "Xiao Yu": "Xiao Yu, what recipes do you recommend after an abortion?" |
| **Week 1** | **Day 4** | **Enabling** | Adequate rest is crucial, but staying in bed all day can hinder recovery. Starting tomorrow, you can take a slow, 5-10 minute walk indoors to promote blood circulation. | Do you know when you can start walking after an abortion and what precautions to take? Let "Xiao Yu" give you some personalized activity advice! |
| **Week 1** | **Day 5** | **Maintaining belief** | Please know that many women have had similar experiences and recovered successfully. This experience is part of life's journey, and the future remains full of hope. | It's normal to have concerns about the future. "Xiao Yu" has some positive recovery stories that might give you strength. Try asking: "Xiao Yu, can you share an encouraging story?" |
| **Week 1** | **Day 6** | **Knowing + Being with** | Monitoring your temperature daily is important. A normal temperature should be below 37.5°C (99.5°F). This is a simple and effective way to check for signs of infection. | Have you checked your temperature today? Does everything feel normal? If you are unsure about anything, you can check with "Xiao Yu" for reassurance. |
| **Week 1** | **Day 7** | **Doing for + Enabling** | Personal hygiene is key. Please wash the external genital area with warm water and change underwear frequently. Avoid baths (use showers) and sexual intercourse for 2 weeks to prevent infection. | Do you have any other questions about personal care, like proper cleansing? Ask "Xiao Yu" for detailed guidance to protect your health. |
| **Week 2** | **Day 8** | **Knowing** | Your hormone levels are gradually returning to normal, which may cause fatigue or low mood. This is a common physiological process. | Have you been feeling particularly tired lately? "Xiao Yu" can explain the scientific reasons behind this to help ease your concerns. |
| **Week 2** | **Day 9** | **Being with** | Emotions need an outlet. Listening to soothing music, journaling, or talking to someone you trust are all great ways to release feelings. | If you don't feel like talking to family or friends, you can always share your thoughts with "Xiao Yu." It's here to offer patient support. |
| **Week 2** | **Day 10** | **Doing for** | If abdominal pain becomes persistent or severe, or if you notice a foul odor in your discharge, it could be a sign of infection. Please seek medical attention promptly. | Are you experiencing any pain or concerns about discharge? Unsure if it's serious? Ask "Xiao Yu" for an initial assessment and advice. |
| **Week 2** | **Day 11** | **Enabling** | If you're recovering well, you can try some gentle exercises, like light yoga or short-distance jogging, but avoid strenuous activities. | Would you like to know what exercises are suitable for you now? Tell "Xiao Yu" your preferences, and it can recommend a suitable recovery exercise plan. |
| **Week 2** | **Day 12** | **Maintaining belief** | You have successfully passed the first week of recovery, which shows your resilience. Please maintain a positive mindset for the better days ahead. | Do you have any hopes or small plans for the future? Share them with "Xiao Yu" – it can share your optimism and cheer you on! |
| **Week 2** | **Day 13** | **Knowing + Enabling** | Your upcoming follow-up examination is very important. It primarily involves an ultrasound and HCG check to ensure the uterus is recovering well without retained tissue. | Are you familiar with the follow-up tests, like the HCG check? If you're unsure why they are needed, ask "Xiao Yu" for an easy-to-understand explanation. |
| **Week 2** | **Day 14** | **Being with + Maintaining belief** | Your follow-up appointment is tomorrow. This is a positive step in your recovery journey. We will be with you. Please relax and attend the appointment as scheduled. | Feeling a bit nervous before the check-up? That's normal. Let "Xiao Yu" encourage you! It can tell you about the process and precautions, supporting you through it. |
